# BitterX: a tool for understanding bitter taste in humans

**DOI:** 10.1038/srep23450

**Published:** 2016-04-04

**Authors:** Wenkang Huang, Qiancheng Shen, Xubo Su, Mingfei Ji, Xinyi Liu, Yingyi Chen, Shaoyong Lu, Hanyi Zhuang, Jian Zhang

**Affiliations:** 1 Institute of Bioinformatics and Medical Engineering, School of Electrical and Information Engineering, Jiangsu University of Technology, Changzhou, 213001, China; 2 Department of Pathophysiology, Key Laboratory of Cell Differentiation and Apoptosis of National Ministry of Education, Shanghai Jiao Tong University, School of Medicine, Shanghai, 200025, China

## Abstract

BitterX is an open-access tool aimed at providing a platform for identifying human bitter taste receptors, TAS2Rs, for small molecules. It predicts TAS2Rs from the molecular structures of arbitrary chemicals by integrating two individual functionalities: bitterant verification and TAS2R recognition. Using BitterX, several novel bitterants and their receptors were predicted and experimentally validated in the study. Therefore, BitterX may be an effective method for deciphering bitter taste coding and could be a useful tool for both basic bitter research in academia and new bitterant discoveries in the industry.

The bitter taste receptors, TAS2Rs, belong to the G protein-coupled receptor super-family and are expressed in the taste receptor cells of taste buds on the gustatory papillae of the tongue. Recently, the extra-oral expression of previously identified TAS2R genes was also found in a variety of tissues^1^,^2^, including gastrointestinal tract^3^, respiratory system^4^, endocrine system^5^, brain^6^, etc. In humans, 25 TAS2Rs are responsible for the recognition of tens of thousands of structurally diverse bitterants. After their initial identification^5^,^7^,^8^, the functional characterization of the receptors was made possible by the advent of expression systems for TAS2Rs^9^,^10^, which led to the rapid deorphanization of the receptors.

To date, 21 of the 25 TAS2Rs have been matched to over 200 bitterants^1^. Among the bitterants identified in recent years, compounds with highly variable identities and structures have been observed, including some bitter-tasting amino acids and peptides^11^,^12^. Interestingly, TAS2Rs use a combinatorial receptor code, in which a certain TAS2R may respond to multiple ligands and a single bitterant may activate multiple TAS2Rs. In addition, some TAS2Rs are broadly tuned but, at the same time, retain exquisite ligand selectivity^1^.

Despite the recent advances in decoding the bitter taste sensation, screening the many natural and synthetic bitter compounds remains a tedious and daunting task; structure-function studies of additional bitterants are still required. To our knowledge, there is no online available tool for predicting ligands for bitter taste receptors in human and vice versa. In this study, we present for the first time a web server tool that can be used to predict the human bitter taste receptors used for certain small molecules. This tool first identifies a bitterant and then predicts its candidate TAS2Rs; it also functions using two individual models aimed at defining a bitterant and then predicting its candidate TAS2Rs. In our benchmark evaluations in the study, the models for bitterant determination and receptor recognition were sufficiently accurate using the test data. More importantly, the TAS2Rs predictions for several bitterants using BitterX were experimentally validated. The dual prediction capability and the user-friendly interface of this web server can be readily utilized in experiments involving TAS2Rs and may serve as a starting point for identifying the respective receptors for chemicals of interest by allowing a more informed approach in selecting both bitterants and their receptors.

## Methods

### Data Collection

Data on the bitterant and bitterant-TAS2R interactions were collected and manually curated from the literature using PubMed and BitterDB^13^. We manually evaluated the curated data to identify bitterant-TAS2R interactions. Taken together, 540 bitterants, including 260 positive and 2379 negative bitterant-TAS2R interactions were collected from the literature and used in this study.

### Physicochemical descriptors

We obtained the molecular structure files for each bitterant from PubChem^14^ and inputted these structures into our in-house program Checker and ChemAxon’s Standardizer (http://www.chemaxon.com). As defined in the Handbook of Molecular Descriptors^15^, 46 and 20 descriptors were selected by Feature Selection (FS) as characteristics of bitterants for the models used in bitterant verification and TAS2R recognition, respectively (Supplementary Tables S1 and S2).

### Receptor descriptors

Using the PseAAC algorithm^16^, with the T-scale properties extracted in a principal components analysis of 67 amino acids^17^, we were able to characterize features of the primary sequences of the TAS2Rs. These features had previously been successfully used to predict cellular protein characteristics and lipase types^16^,^17^,^18^. In the model of TAS2R recognition used here, an optimized set of 15 descriptors, as listed in the Supplementary Table S3, was selected using FS to represent the TAS2Rs.

### SVM classifier

The purpose of Support Vector Machines (SVMs) is to maximize the margin, which is defined as the distance from the separating hyperplane to the closest training samples (support vectors)^19^. Details regarding the theory of SVMs can be found in the literature^19^,^20^. In summary, a given dataset has the corresponding labels of +1 or −1. These values represent the two types of data that are classified as bitter or non-bitter in the bitterant verification and as having a bitterant-TAS2R interaction or no such interaction during TAS2R recognition.

In many supervised learning tasks, it is always necessary to convert the outputs of the classifier into well-calibrated posterior probabilities, particularly when the classification decision is cost-sensitive. Indeed, Platt proposed an SVM + sigmoid method^21^ to estimate the probability of class membership, *f(x*_*i*_), of observation *x*_*i*_. In this study, the probability value of class membership may be interpreted as the probability of bitterant in the model of bitterant verification and the priority of a bitterant binding to its TAS2Rs in the model of TAS2R recognition. A sigmoid function is fitted to all of the estimated *g(x*_*i*_) values to derive probabilities of the following form:


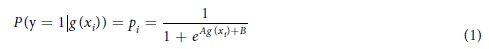


where A and B are estimated by minimizing the negative log-likelihood of the training data:


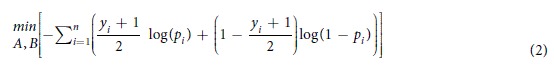


Labels and decision values (estimated *x*_*i*_) are required to be independent, so we conducted a five-fold cross validation (5-CV) to obtain the decision values. The LibSVM package (version 3.17)^22^ was used in this study. To obtain a SVM classifier with optimal performance, the penalty parameter C and the radial basis function (RBF) kernel parameter γ were tuned during FS to obtain SVM classifiers that show optimal performance based on the training set using the grid search strategy in LibSVM.

### Feature selection

FS is a prerequisite because the degree of redundancy and degeneracy for our defined indices may be high. In this study, a Genetic Algorithm (GA) was chosen for its computational efficiency and effectiveness. The general procedures are briefly described as follows:

1. Constant descriptors and descriptors with missing values were first excluded.
2. Highly correlated features were removed, with the correlation coefficient of the filtering threshold set to 0.95.
3. The selection of descriptors and the optimization of SVM parameters were simultaneously performed in the GA. Chromosomes represented both the descriptors and the SVM parameters C and γ in a mixed string mode. The fitness function was designed as a weighted sum of two factors: 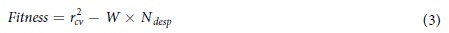
4. where 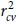 denotes the squared correlation coefficient of a 5-CV in SVM, *N*_*desp*_ represents the number of descriptors used for a chromosome, and *W*(*W* > *0*) is the weight that determines the trade-off between the accuracy and the number of descriptors. Based on our previous experience, *W* was set to 0.007 in this study.
5. For each chromosome in the current generation of the GA, an SVM classifier was trained with its feature subset and SVM parameters. The quality of the chromosome was measured using the classifier’s performance, as evaluated by a 5-CV.
6. In the subsequent selection stage, the fittest chromosomes evolved to the next generation. These steps of evolution continued until the stopping conditions were satisfied.

Throughout this procedure, the feature subset and parameters that gave the best classification performance were selected.

### Performance measurement

As with all discriminative methods^23^,^24^, the performance of statistical learning methods is measured by the quantity of true positives (TP), true negatives (TN), false positives (FP), and false negatives (FN). Precision [PRE = TP/(TP + FP)] is a measure of accuracy for a specific, predicted class. Accuracy [ACC = (TP + TN)/(TP + TN + FP + FN)] is another frequently used index for the overall classification performance, but it may be misleading due to the highly unbalanced class distribution in the used datasets. Sensitivity [SE = TP/(TP + FN)] and specificity [SP = TN/(TN + FP)] can assess a model’s ability to correctly identify TP and TN, respectively, and these two parameters are usually interpreted in combination with each other. All of these four indices were calculated here for model validation and comparison.

In addition to the above four indices, another method that evaluates the tradeoffs between SE and SP in a model is the receiver operating characteristic (ROC) analysis, a performance graphing method that is becoming increasingly important for cost-sensitive learning^25^. The ROC curve typically plots the FP rate (1−SP) versus the TP rate (SE) while varying the decision threshold. The area under the curve (AUC) of the ROC plot provides a convenient way of comparing classifiers, where a random classifier has an area of 0.5 and an ideal classifier has an area of 1.0. In this study, the AUCs were calculated to assess the models.

### Validation of potential TAS2Rs

#### Plasmid construction

The open reading frames of all 25 TAS2Rs were amplified from human genomic DNA and subcloned between the MluI and NotI restriction enzymes sites of Rho-tagged pCI mammalian expression vector (Promega), which was previously constructed to contain the first 20 amino acids of a human rhodopsin tag (N-MNGTEGPNFYVPFSNATGVVR-C)^26^ between the NheI and EcoRI sites. The receptor-transporting proteins, RTP3 and RTP4, which are known to enhance the functional expression of at least some of the bitter taste receptors^27^, were also subcloned into pCI. The chimeric G-protein Gα16/gust44 plasmid was a kind gift from Dr. Takashi Ueda^28^ and was similarly subcloned into pCI. The sequences of the cloned receptors, the receptor-transporting proteins, and the chimeric G-protein were verified by sequencing.

#### Cell culture and immunocytochemistry

Cell culture, live-cell cell-surface immunocytochemistry, and permeablized immunocytochemistry were performed as previously described^29^,^30^,^31^. Briefly, the stock HEK293T cells were cultured in Minimum Essential Medium (HyClone) containing 10% fetal bovine serum (Invitrogen), 500 μg/ml penicillin (HyClone), and 6 μg/ml amphotericin B (Sigma) at 37 °C with 5% CO_2_. To monitor the localization of TAS2Rs to the cell surface, each TAS2R was cotransfected with Gα16/gust44 and RTP3 or RTP4. The RTP constructs were selectively used for the receptors that showed poor cell-surface expression when transfected alone^27^. Eighteen to twenty-four hours after transfection, cells were incubated with the mouse anti-rhodopsin primary antibodies (Millipore) followed by the Alexa Fluor® 555 donkey anti-mouse IgG (Invitrogen) at 4 °C. For permeabilized staining, cells were fixed with 4% paraformaldehyde, permeabilized with 0.2% Triton X-100 at 4 °C, blocked in 5% BSA, and incubated with primary and secondary antibodies. Slides were mounted and visualized using fluoresecence microscopy (Axio Imager A2; Zeiss).

#### Calcium imaging

HEK293T cells were plated in a 96-well plate at a density of 4 × 10^4^ to 5 × 10^4^ cells per well and cotransfected with TAS2Rs and Gα16/gust44 plasmid DNA using Lipofectamine2000 (Invitrogen). Eighteen to twenty-four hours post-transfection, the media were removed and the cells were loaded with the calcium-sensitive dye, Fluo-4-NW (Molecular Probes), for 30 min at 37 °C followed by an additional 30 min at room temperature. In our study, Fluo-4-NW was dissolved in 20 mM HEPES to a final concentration of 2 μM; this solution also contained 2.5 mM Probenecid (Invitrogen), an inhibitor of organic anion transport. Next, 100 μl of this dye-loading solution was added to each well of a 96-well plate. After loading, 20 μl of the various concentrations of the compound of interest was added to the wells, and fluorescence was measured using a FDSS/μCELL plate reader (Hamamatsu) every second for 180 seconds.

#### Experimental study of TAS2R recognition

A total of 5 compounds were tested against their predicted TAS2R hits at concentrations of 3 and 30 mM and with three replicates. The receptors that gave a positive result in the preliminary testing were further tested at 8–12 different concentrations. Dose-response curves were generated, and the half-maximal effective concentration (EC_50_) values were calculated by nonlinear regression using GraphPad PRISM 5.0 with the equation:


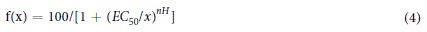


where x is the agonist concentration and nH is the Hill coefficient.

## Results

### Bitterant verification

**Figure 1:**
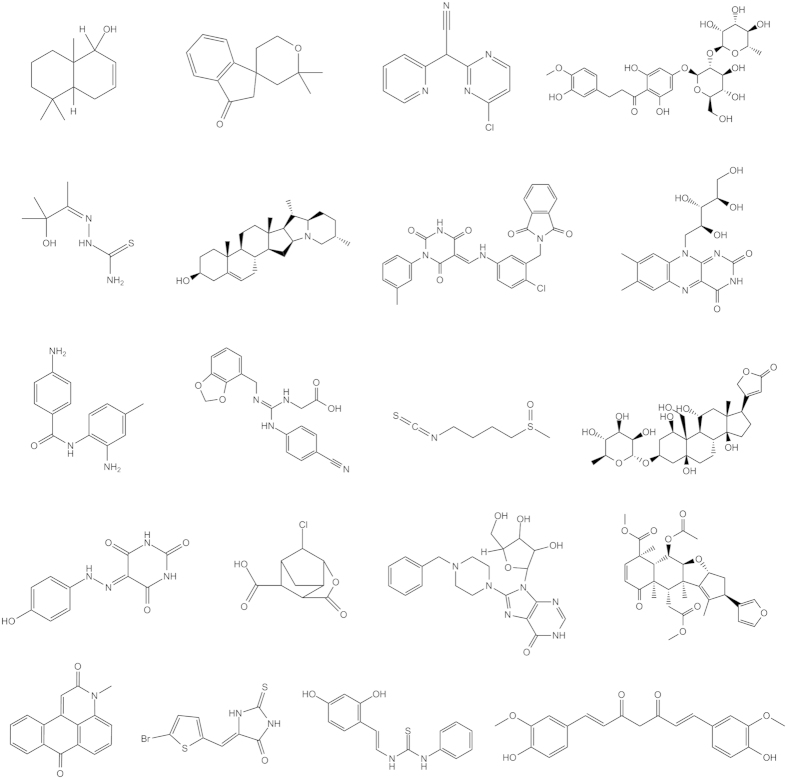
Structures of 20 true bitterless compounds from in-house experimental validation.

Among the 540 bitterants in BitterX, the procedures described in physicochemical descriptors were followed to generate the descriptors. After the removal of a failed compound, 539 bitterants were retained as the positive set that was used for model building. The total number of negatives should equal that of the positives. First, 20 true non-bitterants from in-house experimental validation were added into the negative set (Fig. 1). Second, pseudo non-bitterants were obtained from a large chemical database, known as the Available Chemicals Directory (ACD, http://www.accelrys.com), in which all compounds without the label “Bitter” were clustered into 519 classes using the fingerprint encoded in Openbabel (http://openbabel.org). The compound in each cluster center was extracted to add into the negative set representing non-bitterants. The positive and negative sets were then combined into the final dataset, which consisted of 1078 entries that were used to build a classification model for bitterants.

To train a classification model for bitterants, 862 molecules (80% of the dataset), half of which were positives and half negatives, were randomly selected from the full dataset, and both descriptors and parameters for the model were optimized by FS. As a result, 46 descriptors were selected (Supplementary Table S1), and the optimal SVM parameters C and γ were 32 and 0.015625, respectively. Next, the 216 remaining molecules in the dataset (20% of the dataset) were treated as a test set and used for predictions in the SVM model, which was built from the 862 molecules in the training data set using the aforementioned descriptors and optimal parameters. In addition, another two sets of training and test data with the same number of molecules were randomly partitioned to build and test our method to avoid any potential bias in the data splitting. The overall results are shown in Table 1. For both the 5-CV and test set predictions, the specificity, sensitivity and precision were above 90%, the prediction accuracy was above 87% and the AUC was above 94%. On average, our method produced a high-quality classification model for the prediction of bitterants. Finally, 1078 chemicals were used to build the final bitterant classification model using the same descriptors and optimal parameters; as a result, the first model was deployed on the BitterX web server.

### TAS2R recognition

To assemble a classification model for TAS2R recognition, a rational dataset, including both positive and negative bitterant-TAS2R pairs was essential. The known TAS2R database contains 260 non-redundant entries of experimentally verified bitterant-TAS2R interactions that were obtained from a manual search of the literature. These bitterant-TAS2R pairs were all used in the positive dataset. Selecting an appropriate negative dataset was essential for the reliability of the prediction model. However, it was difficult to generate this dataset because there were more negative data than positive data in the real world. Furthermore, we have limited information regarding the bitterant-TAS2R pairs that were truly non-interactive. Instead of randomly selecting entries for the negative dataset, we used a relatively rational strategy that considered whether an TAS2R appeared in the positive dataset. If an TAS2R was shown as both a positive and negative bitterant-TAS2R pair in our collection, the entries of the negative TAS2R pair were moved to the negative dataset. Otherwise, pseudo-negative bitterant-TAS2R pairs for a given TAS2R would be established by mismatching a positive bitterant-TAS2R pair. Additionally, the contribution of the TAS2Rs in the negative dataset should be as balanced as possible. In total, 260 positive and 260 negative bitterant-TAS2R pairs were obtained to create the final dataset for building a classification model for TAS2R recognition (Supplementary Tables S4 and S5).

To assemble a classification model for TAS2R recognition, the dataset was randomly divided into the training and test sets at a 4:1 ratio; the descriptors for both bitterants and receptors as well as the parameters for the SVM model were optimized for the training set using the FS procedure. Thirty-five descriptors, including 20 physicochemical and 15 receptor features, were selected from GA, and the optimal SVM parameters C and γ were derived to be 8.0 and 0.125, respectively. Subsequently, an SVM model based on the selected descriptors and parameters was constructed to predict the test set. Similar to the building of the bitterant verification model, another two sets of training and test data with the same number of pairs were randomly partitioned to build and test the second SVM model. The overall performances of the SVM models are provided in Table 2. For both the 5-CV and test set predictions, the prediction accuracy was above 76%, the specificity and precision were above 75%, the sensitivity was above 78%, and the AUC was above 81%. Finally, the entire dataset was used to construct a probability-based classification model for TAS2R recognition using the descriptors and optimal parameters, and it was deployed on the BitterX web server as the second model.

### Evaluation of Models in BitterX

We have extensively evaluated our algorithm for both bitterant verification and receptor recognition, and the results are shown in Tables 1 and 2; four additional examples of these are provided on the Help page of the BitterX server. The accuracy values for the test set predictions indicate that our models have satisfactory prediction capabilities for discovering bitterants and their receptors. To further test the reliability of the BitterX server in practice, potential compounds from an in-house fragment library, which is composed of more than 220 small organic compounds and is used in fragment-based hit discovery, were screened using the BitterX server for their bitterness response among the TAS2Rs (Supplementary Table S6). Among these compounds, five were predicted to be bitter, and their potential receptors were determined.

**Figure 2:**
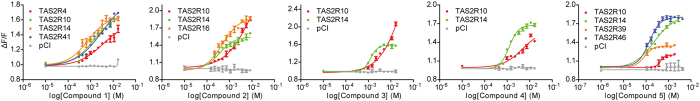
The dose-response curves of all 15 bitterant-TAS2R interactions listed in Table 3. *y*-axis represents ΔF/F ± SEM (*N *= 2). “pCI” denotes the response of mock-transfected cells to each compound.

Furthermore, the interactions between the five compounds and their potential receptors as identified via BitterX were evaluated using a calcium imaging assay. The results showed that a total of 7 TAS2Rs were experimentally validated for the five compounds (Fig. 2 and Table 3). (For TAS2R expression, see Supplementary Figure S1) Therefore, we believe that our computational method represents an effective method for deciphering bitter taste coding. It may also be a useful tool for both basic bitterant screening and industry-scale TAS2R identification.

### Usage and output

**Figure 3:**
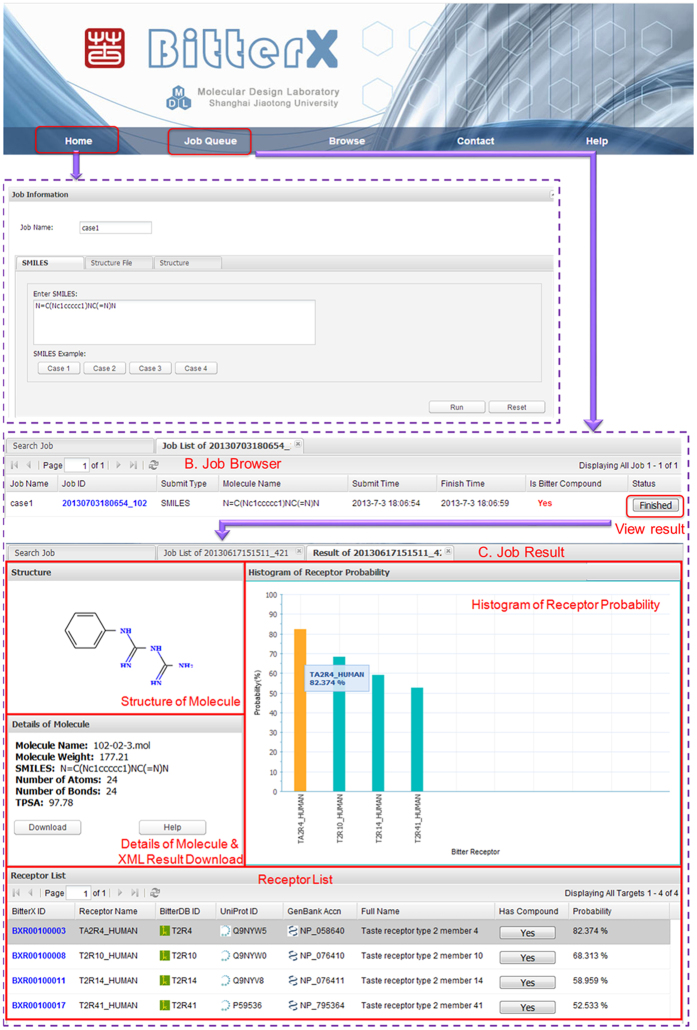
The web interface and output of BitterX. (**A**) Query input in homepage (**B**) Bitterant-TAS2R interaction entries in “Browse” page. (**C**) An example of an output page after submitting a chemical molecule. A confidence score in probability is displayed along with the associated TAS2R in both the “Receptor List” and the Column Chart, which can be retrieved by clicking “Show Receptor Histogram”.

The Bitterant Identification functionality in BitterX performs two steps, a bitterant verification and TAS2R recognition, which are extensively described in the Materials and Methods. Three types of inputs need to be provided for the organic molecule query: entering the SMILES under “SMILES”, uploading a molecular file in the MDL mol format under “Structure File”, and sketching the structure under “Structure”. After defining the query chemicals, a mandatory parameter, “Job Name”, must be set to enable the users to easily monitor their queries in BitterX. Once a run is submitted, a transition window with a unique Job ID based on the current date and time appears and serves as a permanent bookmarkable link to the data. The users may use the Job ID or Job Name to track the progress of the calculations on the BitterX “Job Queue” page. A typical run of BitterX takes 5–10 seconds, depending on the complexity of the input chemical. Therefore, large molecules with more than 200 atoms were not tested in the current version of BitterX because of the extended running time. When a job is completed, a button labeled “Finished” emerges on the “Job Queue” page and redirects the users to the results (Fig. 3).

The output of BitterX is split into four main sections: “Structure”, “Receptor List”, “Histogram of Receptor Probability” and “Details of Molecule”. If the query molecule shown in the “Structure” section is predicted to be positive in the bitterant verification, the subsequent TAS2R recognition is automatically run to infer the bitterant’s potential receptors, which are then ranked according to the probability scores from the “Receptor List” section and compared to one another in the section “Histogram of Receptor Probability”. The score ranges from 0% to 100%, with 0% indicating maximum confidence for non-interactions and 100% indicating maximum confidence for an interaction between the bitterant and an TAS2R. For example, a potential TAS2R for a query molecule with an estimated probability score of 90% should be more likely to be the correct receptor than an TAS2R with a probability score of 60%. In addition to the visualization of the results, all results and annotations in BitterX can be downloaded as an XML text file for additional analysis on the “Details of Molecule” panel.

## Discussion

Over the past few years, the functions of TAS2Rs have been expanded from their canonical gustatory function. Novel TAS2R functions include the respiratory and gastrointestinal responses that are associated with metabolic and digestive regulation. Indeed, additional physiological functions may be ascribed to the TAS2Rs and their respective ligands^31^. However, due to the tremendous size of the chemical space, identifying ligand spectra and their structure-function relationships with TAS2Rs remains a challenge, with 4 of 25 the TAS2Rs remaining orphaned^1^. Here, we describe an open access tool, the BitterX web service that developed for the recognition of chemical bitterants and their cognate TAS2Rs. The program’s ability to identify novel bitterants and their TAS2Rs was successfully demonstrated using simulated validations and experimental screening. To the best of our knowledge, this web server is the first of its type and may be of considerable value to bitter taste research. In the future, to ensure the strength of this web service, we will continue to integrate into the dataset up-to-date information from the literature of both bitterants and their TAS2Rs to improve the predictive power of the methods. The BitterX server is available at http://mdl.shsmu.edu.cn/BitterX.

## Additional Information

**How to cite this article**: Huang, W. *et al*. BitterX: a tool for understanding bitter taste in humans. *Sci. Rep.* **6**, 23450; doi: 10.1038/srep23450 (2016).

## Supplementary Material

Supplementary Information

## Figures and Tables

**Table 1:** The performance of the SVM model in the prediction of bitterants.

Exp.	Training set (5-CV)				Test set							
	Pos.	Neg.	Sum.	ACC	Pos.	Neg.	Sum	SE	SP	PRE	ACC	AUC
1	431	431	862	0.8828	108	108	216	0.9083	0.9159	0.9167	0.9120	0.9421
2	431	431	862	0.8805	108	108	216	0.9174	0.9065	0.9091	0.9120	0.9489
3	431	431	862	0.8736	108	108	216	0.9358	0.9065	0.9107	0.9213	0.9589

**Table 2:** The performance of the SVM model in the prediction of the bitterant-TAS2R interactions.

Exp.	Training set (5-CV)				Test set							
	Pos.	Neg.	Sum.	ACC	Pos.	Neg.	Sum	SE	SP	PRE	ACC	AUC
1	209	209	418	0.7644	51	51	102	0.8269	0.7692	0.7818	0.7981	0.8291
2	209	209	418	0.7620	51	51	102	0.7885	0.8269	0.8200	0.8077	0.8088
3	209	209	418	0.7740	51	51	102	0.8269	0.7500	0.7679	0.7885	0.8306

**Table 3:**
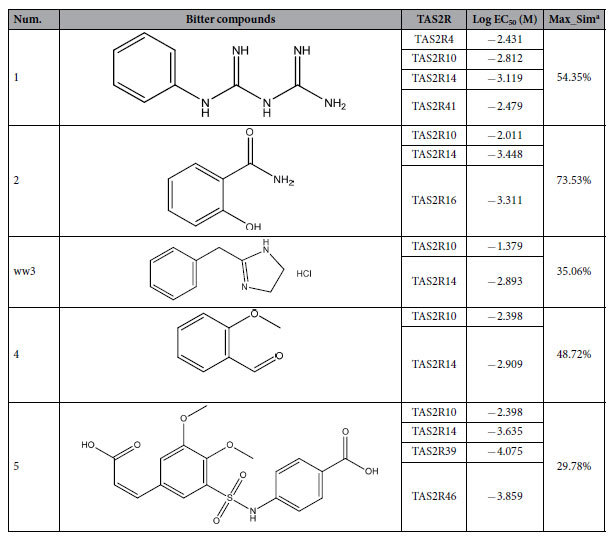
Experimentally validated bitterant-TAS2R interactions are shown with the EC_50_ values and maximum structural similarities.

^a^Maximum structural similarities of the screened compounds with the respective existing bitterants of the TAS2Rs have been calculated by using the FP2 method encoded in Openbabel (http://openbabel.org), which is a path-based fingerprint indexes on small molecule fragments.
