# Supplementary material for: BitterX: a tool for understanding bitter taste in humans
Source: Sci Rep. 2016 Apr 4;6:23450. doi: 10.1038/srep23450 (PMC4819188; doi:10.1038/srep23450)
Supplement: Supplementary Information [file srep23450-s1.pdf]

Supplementary Data

BitterX: a tool for understanding bitter taste in humans

Wenkang Huang[1,2,||], *Qiancheng Shen*[2,||], *Xubo Su*[2,||], *Mingfei Ji*[2,||], *Xinyi Liu*[2], *Yingyi Chen*[2], *Shaoyong Lu*[2], *Hanyi Zhuang*[2,*], *Jian Zhang*[1,2,*]

[1] Institute of Bioinformatics and Medical Engineering, School of Electrical and Information Engineering, Jiangsu University of Technology, Changzhou, 213001, China

[2] Department of Pathophysiology, Key Laboratory of Cell Differentiation and Apoptosis of National Ministry of Education, Shanghai Jiao Tong University, School of Medicine, Shanghai, 200025, China.

*Correspondence to: Jian Zhang (Phone: +86-21-63846590-776922; Fax: +86-21-64154900; Email: jian.zhang@sjtu.edu.cn) or Hanyi Zhuang (Email: hanyizhuang@sjtu.edu.cn)

[||]W. Huang, Q. Shen, X. Su and M. Ji contributed equally to this work.

1 TABLES

Table S1. The 46 physicochemical descriptors used in the model of bitterant estimation. Definitions of how the descriptors were calculated can be found in the *Handbook of Molecular Descriptors* (Todeschini and Consonni, 2000).

Num.	Physicochemical Property
1	Average connectivity index chi-1
2	Binary 2 order of C and O
3	Binary 4 order of C and O
4	Binary 5 order of C and C
5	Binary 5 order of C and N
6	Binary 7 order of C and O
7	Binary 8 order of C and C
8	Broto-Moreau autocorrelation of a topological structure - lag 7 / weighted by atomic van der Waals volumes
9	Complementary Information Content 0
10	Complementary Information Content 4
11	Conventional bond-order ID number
12	Difference between multiple path count and path count
13	First Mohar index
14	Frequency 2 order of C and N
15	Frequency 2 order of C and O
16	Frequency 4 order of C and C
17	Frequency 9 order of C and O
18	Geary autocorrelation - lag 1 / weighted by atomic polarizabilities
19	Geary autocorrelation - lag 4 / weighted by atomic Sanderson electronegativities
20	Geary autocorrelation - lag 6 / weighted by atomic masses
21	Geary autocorrelation - lag 6 / weighted by atomic Sanderson electronegativities
22	Geary autocorrelation - lag 7 / weighted by atomic masses
23	Geary autocorrelation - lag 8 / weighted by atomic masses
24	Information content index 0
25	Kier&Hall order 1
26	MACCS No 96
27	MACCS No 112
28	MACCS No 131
29	MACCS No 139
30	MACCS No 149
31	MACCS No 153
32	MACCS No 157
33	MACCS No 158
34	MACCS No 159

35	MACCS No 164
36	Maximal electrotopological negative variation
37	Mean topological charge index of order 4
38	Mean topological charge index of order 7
39	Molecular walk count of order 3
40	Moran autocorrelation - lag 2 / weighted by atomic Sanderson electronegativities
41	Moran autocorrelation - lag 5 / weighted by atomic Sanderson electronegativities
42	O atom num
43	Rel C atom num
44	Ring num of member 5
45	Structural information content 2
46	Total information content index 0

Table S2. The 20 physicochemical descriptors used in the model of hTAS2R recognition. Definitions of how the descriptors were calculated can be found in the *Handbook of Molecular Descriptors* (Todeschini and Consonni, 2000).

Num.	Physicochemical Property
1	Average connectivity index chi-3
2	Broto-Moreau autocorrelation of a topological structure - lag 7 / weighted by atomic polarizabilities
3	Broto-Moreau autocorrelation of a topological structure - lag 8 / weighted by atomic polarizabilities
4	Broto-Moreau autocorrelation of a topological structure - lag 8 / weighted by atomic masses
5	Frequency of C-C at topological distance 6 (frequency 6 order of C and C)
6	Frequency of C-C at topological distance 7 (frequency 7 order of C and C)
7	Geary autocorrelation - lag 1 / weighted by atomic Sanderson electronegativities
8	Geary autocorrelation - lag 2 / weighted by atomic Sanderson electronegativities
9	Geary autocorrelation - lag 3 / weighted by atomic masses
10	Geary autocorrelation - lag 7 / weighted by atomic masses
11	Mean atomic polarizability (Average Pol)
12	Mean topological charge index of order 2
13	Mean topological charge index of order 3
14	Relative O atom number
15	Moran autocorrelation - lag 2 / weighted by atomic polarizabilities
16	Moran autocorrelation - lag 3 / weighted by atomic Sanderson electronegativities

17	Moran autocorrelation - lag 5 / weighted by atomic polarizabilities
18	Self returning walk of order 10
19	Structural information content 3
20	Valence average connectivity index chi-6

Table S3. The 15 receptor descriptors used in the model of hTAS2R recognition. Definitions of how the descriptors were calculated can be found in Chou, 2001 and Tian *et al.*, 2007.

Num.	Physicochemical Property
1	Amino Acid Hydropathy Score
2	Normalized frequency of amino acid A
3	Normalized frequency of amino acid F
4	Normalized frequency of amino acid S
5	Normalized frequency of amino acid V
6	Normalized frequency of amino acid Y
7	Pseudo amino acid θ_3
8	Pseudo amino acid θ_{11}
9	Pseudo amino acid θ_{18}
10	Pseudo amino acid θ_{25}
11	Pseudo amino acid θ_{26}
12	Pseudo amino acid θ_{34}
13	Pseudo amino acid θ_{37}
14	Pseudo amino acid θ_{38}
15	Pseudo amino acid θ_{44}

Table S4. Positive bitterant-TAS2R pairs used in the TAS2R recognition model.

Molecule	Target
CC1=CC(O)C2C(CC(=C)C(CC1)O)OC(=O)C2=C	TAS2R46
CN(CCOC(c1ccccc1C)c1ccccc1)C	TAS2R46
Oc1cc2O[C@H](c3ccc(c(c3)O)O)[C@@H](Cc2c(c1)O)O	TAS2R5,TAS2R39
Oc1cc2O[C@H](c3cc(O)c(c(c3)O)O)[C@@H](Cc2c(c1)O)O	TAS2R39
Oc1cc(O)c2c(c1)O[C@@H]([C@@H](C2)OC(=O)c1cc(O)c(c(c1)O)O)c1ccc(c(c1)O)O	TAS2R39
OC[C@H]1O[C@@H](Oc2cc(O)c3c(c2)occ(c3=O)c2ccc(cc2)O)[C@@H]([C@H]([C@@H]1O)O)O	TAS2R39
NC(=S)Nc1ccccc1	TAS2R38
CN1CCN(CC1)c1c(F)cc2c3c1OCC(n3cc(c2=O)C(=O)O)C	TAS2R9
CCN(CCNC(=O)c1ccc(cc1)N)CC	TAS2R9
CN1CCN(CC1)CC(=O)N1C2CCCCC2C(=O)NC2C1NCCC2	TAS2R9
CCCc1cc(=O)[nH]c(=S)[nH]1	TAS2R38

CC(=O)Nc1ccc(cc1)O	TAS2R39
CC1=CC(=NS(=O)(=O)O1)[O-]	TAS2R44,TAS2R43
OCc1cc(O)c2c(c1)C(C1OC(CO)C(C(C1O)O)O)c1c(C2=O)c(O)ccc1	TAS2R44,TAS2R43
CC1C(=O)CC2C1C1OC(=O)C(=C)C1C(CC2=C)O	TAS2R46,TAS2R43
O=C1NS(=O)(=O)c2c1cccc2	TAS2R44,TAS2R43
OCC1(C)C(O)CCC2(C1CCC(=C)C2C/C=C/1\C(O)COC1=O)C	TAS2R46,TAS2R47,TAS2R50
OCC1=CC2OC(=O)C(=C)C2C(CC(=CCC1)C)OC(=O)C(=C)C(CO)O	TAS2R46
OOC1CCC2(CC1=CCC1C(C2O)OC(=O)C1=C)C	TAS2R46
OCC(=O)C1(O)CCC2C1(C)CC(O)C1C2CCC2=CC(=O)CCC12C	TAS2R46
S=C=NCCc1ccccc1	TAS2R38
N/C(=N\CCCCCC/N=C(/N=C(/Nc1ccc(cc1)Cl)\N)\N)/N=C(/Nc1ccc(cc1)Cl)\N	TAS2R14
CCCC(COC(=O)N)(COC(=O)NC(C)C)C	TAS2R14
COC1=CC(C)C2C(C1=O)(C)C1C(=O)C(=C(C3C1(C(C2)OC(=O)C3)C)C)OC	TAS2R46,TAS2R47,TAS2R4,TAS2R10,TAS2R14
CC1=CCCC2(C)OC2C2C(CC1)C(=C)C(=O)O2	TAS2R46,TAS2R44,TAS2R1,TAS2R4,TAS2R8,TAS2R10,TAS2R14
NC(=O)c1ccccc1	TAS2R14
Cn1cnc(c1Sc1ncnc2c1[nH]cn2)[N+](=O)[O-]	TAS2R46,TAS2R4,TAS2R10,TAS2R14
CCCCCCC/C=C/C(C#CC#CC(C=C)O)O	TAS2R43,TAS2R14
CC(=O)OC(/C=C/C(=O)C(C1C(O)CC2(C1(C)CC(=O)C1(C2CC=C2C1CC(O)C(=O)C2(C)C)C)C)(O)C)(C)C	TAS2R10,TAS2R14
COc1ccc(cc1OC)Cc1nccc2c1cc(OC)c(c2)OC	TAS2R7,TAS2R10,TAS2R14
COc1ccc2c(c1OC)C(=O)OC2C1N(C)CCc2c1c(OC)c1c(c2)OCO1	TAS2R14
Oc1ccc2c(c1)oc(=O)c1c2oc2c1ccc(c2)O	TAS2R39,TAS2R14
S=C1NCCN1C	TAS2R38
O=C1OCC23C(C1)OC(C2CC(=O)C1(C3CCC2(C31OC3C(=O)OC2c1cocc1)C)C)(C)C	TAS2R38
CNC(=S)N	TAS2R38
CC(=C)C1C2OC(=O)C1C1(C3(C2OC(=O)C23C(C1)O2)C)O	TAS2R46,TAS2R47,TAS2R1,TAS2R10,TAS2R14
CCc1cnccn1	TAS2R38
S=CN(C)C	TAS2R38
S=C1NCCN1	TAS2R38
CCNC(=S)N	TAS2R38
CC(=CCC(=O)C1(O)C(=C(C(=O)C1CC=C(C)C)C(=O)C(C)C)O)C	TAS2R1,TAS2R14
CC(=CCC(=O)C1(O)C(=C(C(=O)C1CC=C(C)C)C(=O)C(C)C)O)C	TAS2R1,TAS2R14
CCC(C(=O)C1=C(O)C(C(=O)C(=C1O)CC=C(C)C)(CC=C(C)C)CC=C(C)C)C	TAS2R1,TAS2R14
[O-]S(=O)(=O)NC1CCCCC1	TAS2R1,TAS2R38
CCN(CCCC(Nc1ccnc2c1ccc(c2)Cl)C)CC	TAS2R3,TAS2R10,TAS2R39,TAS2R14

CCC(C(=O)C1=C(O)C(C(C1=O)CC=C(C)C)(O)C(=O)CC=C(C)C)C	TAS2R1,TAS2R14
CCC(C(=O)C1=C(O)C(C(C1=O)CC=C(C)C)(O)C(=O)CC=C(C)C)C	TAS2R1,TAS2R14
CC(=O)NC(=S)N	TAS2R38
O=C1CCCCCN1	TAS2R38
OCC1OC(OCC2OC(OC(c3ccccc3)C#N)C(C(C2O)O)O)C(C(C1O)O)O	TAS2R16
OCC1OC(Oc2ccc(cc2)O)C(C(C1O)O)O	TAS2R16
O=C1CC2OCC=C3C4C2C2N1c1ccccc1C12CCN(C1C4)C3	TAS2R46,TAS2R10
NC(=Nc1scc(n1)CSCC/C(=N/S(=O)(=O)N)/N)N	TAS2R44,TAS2R10
C=CC/C(=N\OS(=O)(=O)[O-])/SC1OC(CO)C(C(C1O)O)O	TAS2R38,TAS2R16
C=CCN=C=S	TAS2R38
OCC1OC(Oc2ccccc2C=O)C(C(C1O)O)O	TAS2R43,TAS2R16
OCC1OC(Oc2ccccc2CO)C(C(C1O)O)O	TAS2R16
C=CC1CN2CCC1CC2C(c1ccnc2c1cc(OC)cc2)O	TAS2R46,TAS2R44,TAS2R43,TAS2R40,TAS2R4,TAS2R7,TAS2R10,TAS2R39,TAS2R14
CC(=CCC1=C(O)C(C(=O)C(=C1O)C(=O)CC(C)C)(CC=C(C)C)CC=C(C)C)C	TAS2R1,TAS2R14
OC(C(=O)c1ccccc1)c1ccccc1	TAS2R10,TAS2R14
CC(=CCC1=C(O)C(C(=O)C(=C1O)C(=O)CC(C)C)(O)CC=C(C)C)C	TAS2R40,TAS2R1
OCC1OC(Oc2ccccc2)C(C(C1O)O)O	TAS2R16
CN(CCC(c1ccccn1)c1ccc(cc1)Cl)C	TAS2R46,TAS2R40,TAS2R4,TAS2R7,TAS2R10,TAS2R39,TAS2R38,TAS2R14
CN(CCOC(c1ccccc1)c1ccccc1)C	TAS2R14
OC(c1ccccc1)(c1ccccc1)CCCN1CCCCC1	TAS2R49,TAS2R46,TAS2R47,TAS2R44,TAS2R43,TAS2R40,TAS2R1,TAS2R4,TAS2R7,TAS2R13,TAS2R10,TAS2R39,TAS2R38,TAS2R14,TAS2R16
S=C(Nc1ccccc1)Nc1ccccc1	TAS2R1,TAS2R38,TAS2R14
C=CS(=O)C=C	TAS2R14
OC(=O)c1ccccc1Nc1cccc(c1)C(F)(F)F	TAS2R14
Fc1ccc(cc1)C(=O)CCCN1CCC(CC1)(O)c1ccc(cc1)Cl	TAS2R10,TAS2R14
[O-]C(=O)c1ccccc1	TAS2R14,TAS2R16
OCCc1sc[n+](c1C)Cc1cnc(nc1N)C	TAS2R1,TAS2R39
OCC(C(c1ccc(cc1)[N+](=O)[O-])O)NC(=O)C(Cl)Cl	TAS2R46,TAS2R43,TAS2R1,TAS2R8,TAS2R10,TAS2R39,TAS2R41
OCC1OC(Oc2ccccc2CO)C(C(C1O)O)O	TAS2R16
CC1CC(C)C(=O)C(C1)C(CC1CC(=O)NC(=O)C1)O	TAS2R10
COc1cc2c(cc1OC)N1C3C42CCN2C4CC4C3C(CC1=O)OCC=C4C2	TAS2R46,TAS2R4
O=C1CC2(C(C1C)C2)C(C)C	TAS2R10,TAS2R14
Nc1ccc(cc1)S(=O)(=O)c1ccc(cc1)N	TAS2R40,TAS2R4,TAS2R10
COc1c(OC)cc2c(c1OC)c1ccc(c(=O)cc1C(CC2)NC(=O)C)OC	TAS2R46,TAS2R4,TAS2R39

c1ccc2c(n1)c1ncccc1cc2	TAS2R5
CC[N+](Cc1ccccc1)(CC(=O)Nc1c(C)cccc1C)CC	TAS2R46,TAS2R47,TAS2R43,TAS2R4,TAS2R8,TAS2R13,TAS2R10,TAS2R39
CC(=O)OC(/C=C/C(=O)C(C1C(O)CC2(C1(C)CC(=O)C1(C2CC=C2C1C=C(O)C(=O)C2(C)C)C)C)(O)C)(C)C	TAS2R10
OC(COc1cccc2c1c(=O)cc(o2)C(=O)O)COc1ccc2c(c1)c(=O)cc(o2)C(=O)O	TAS2R49,TAS2R43,TAS2R7
CC(=CCc1c(O)cc(c2c1OC(CC2=O)c1ccc(cc1)O)O)C	TAS2R14
Cc1cc(O)nc(n1)S	TAS2R38
Oc1cc(O)c2c(c1)O[C@@H]([C@H]([C@@H]2c1c(O)cc(c2c1O[C@H](c1ccc(c(c1)O)O)[C@H]([C@@H]2c1c(O)cc(c2c1O[C@@H]([C@H](C2)O)c1ccc(c(c1)O)O)O)O)O)O)c1ccc(c(c1)O)O	TAS2R5
C[C@@H]1C(=O)O[C@H]2[C@H]1CC[C@@]1(C2=C(C)C(=O)C=C1)C	TAS2R10
OC[C@H]1O[C@@H](Oc2cc3c(O)cc(cc3[o+]c2c2cc(OC)c(c(c2)OC)O)O)[C@@H]([C@H]([C@@H]1O)O)O	TAS2R5,TAS2R7
O=C(c1cc(O)c(c(c1)O)O)O[C@@H]1[C@@H](OC(=O)c2cc(O)c(c(c2)O)O)[C@@H](OC([C@@H]1OC(=O)c1cc(O)c(c(c1)O)O)OC(=O)c1cc(O)c(c(c1)O)O)OC(=O)c1cc(O)c(c(c1)O)O	TAS2R39
O=CC1(CC(c2cocc2)O)C(C)C(OC(=O)C)CC2(C1CCC(C2(C)O)O)C	TAS2R46,TAS2R47,TAS2R1,TAS2R10,TAS2R14
O=c1ccc2c(o1)cccc2	TAS2R10,TAS2R14
Cn1cnc2c1c(=O)n(C)c(=O)n2C	TAS2R46,TAS2R43,TAS2R7,TAS2R10,TAS2R14
O=C1OC(=O)C2(C(C1CC2)(C)C)C	TAS2R47,TAS2R4,TAS2R10,TAS2R14
COc1cccc2c1cc([N+](=O)[O-])c1c2c2OCOc2cc1C(=O)O	TAS2R44,TAS2R43,TAS2R14
CC1=CC2OC(=O)C(=C)C2CCC(=C)C(CC1)O	TAS2R46,TAS2R47,TAS2R4,TAS2R10,TAS2R14
CC1=CCC23C1C1OC(=O)C(C1CCC3(O2)C)C	TAS2R46,TAS2R43,TAS2R1,TAS2R4,TAS2R10,TAS2R14
CC1=CCC23C1C1OC(=O)C(=C)C1CCC3(O2)C	TAS2R46,TAS2R43,TAS2R10,TAS2R14
CC1C(=O)OC2C1CCC(C1C2=C(C)C2C1C1C3C2(C(=C1)C)C1OC(=O)C(C1CCC3(C)O)C)(C)O	TAS2R46,TAS2R47,TAS2R10,TAS2R14
COc1ccc2c(c1)C13CCCCC3C(C2)N(CC1)C	TAS2R1,TAS2R10
COc1cc(O)c(c(c1C(=O)/C=C/c1ccc(cc1)O)O)CC=C(C)C	TAS2R40,TAS2R1,TAS2R14
COc1cc(O)c(c2c1C(=O)CC(O2)c1ccc(cc1)O)CC=C(C)C	TAS2R40,TAS2R1,TAS2R14
CC(=CCC1=C(O)C(C(=O)C(=C1O)C(=O)C(C)C)(CC=C(C)C)CC=C(C)C)C	TAS2R40,TAS2R1
CC(=CCC1=C(O)C(C(=O)C(=C1O)C(=O)C(C)C)(O)CC=C(C)C)C	TAS2R40,TAS2R1
CCC(C(=O)C1=C(O)C(C(=O)C(C1=O)CC=C(C)C)(O)CC=C(C)C)C	TAS2R40,TAS2R1
OCC1OC(OC2OC=C3C(C2C=C)CCOC3=O)C(C(C1O)O)OC(=O)c1c(O)cc(cc1c1cccc(c1)O)O	TAS2R46,TAS2R47,TAS2R43,TAS2R1,TAS2R4,TAS2R39,TAS2R50

COC(=O)C1C(O)CCC2C1CC1N(C2)CCc2c1[nH]c1c2cccc1	TAS2R46,TAS2R1,TAS2R4,TAS2R10,TAS2R38
CC(CC(=O)C1=C(O)C(C(C1=O)CC=C(C)C)(O)C(=O)CC=C(C)C)C	TAS2R1,TAS2R14
Oc1cc(O)c2c(c1)O[C@@H]([C@@H](C2)OC(=O)c1cc(O)c(c(c1)O)O)c1cc(O)c(c(c1)O)O	TAS2R39
Oc1ccc(cc1)[C@H]1COc2c(C1)ccc(c2)O	TAS2R39,TAS2R14
Oc1ccc(cc1)c1coc2c(c1=O)c(O)cc(c2)O	TAS2R39,TAS2R14

Table S5. Negative bitterant-TAS2R pairs used in the TAS2R recognition model.

Molecule	Target
CC1=CC(O)C2C(CC(=C)C(CC1)O)OC(=O)C2=C	TAS2R43, TAS2R47, TAS2R9
CN(CCOC(c1ccccc1C)c1ccccc1)C	TAS2R48, TAS2R8, TAS2R60
OCC1OC(OCC2OC(O)C(C(C2O)O)O)C(C(C1O)O)O	TAS2R1
Oc1cc2O[C@H](c3ccc(c(c3)O)O)[C@@H](Cc2c(c1)O)O	TAS2R4
Oc1cc2O[C@H](c3cc(O)c(c(c3)O)O)[C@@H](Cc2c(c1)O)O	TAS2R47
Oc1cc(O)c2c(c1)O[C@@H]([C@@H](C2)OC(=O)c1cc(O)c(c(c1)O)O)c1ccc(c(c1)O)O	TAS2R1, TAS2R16, TAS2R4
OC[C@H]1O[C@@H](Oc2cc(O)c3c(c2)occ(c3=O)c2ccc(cc2)O)[C@@H]([C@H]([C@@H]1O)O)O	TAS2R43
CCN(CCCC(Nc1ccnc2c1ccc(c2)Cl)C)CC	TAS2R60, TAS2R5, TAS2R49
CN1CCN(CC1)c1c(F)cc2c3c1OCC(n3cc(c2=O)C(=O)O)C	TAS2R42, TAS2R46
CCN(CCNC(=O)c1ccc(cc1)N)CC	TAS2R13, TAS2R43, TAS2R46
CN1CCN(CC1)CC(=O)N1C2CCCCC2C(=O)NC2C1NCCC2	TAS2R8, TAS2R43, TAS2R10
CCCc1cc(=O)[nH]c(=S)[nH]1	TAS2R60, TAS2R50, TAS2R16
CC(=O)Nc1ccc(cc1)O	TAS2R9, TAS2R7, TAS2R16
CC1=CC(=NS(=O)(=O)O1)[O-]	TAS2R39, TAS2R40
OCc1cc(O)c2c(c1)C(C1OC(CO)C(C(C1O)O)O)c1c(C2=O)c(O)ccc1	TAS2R1, TAS2R3
CC1C(=O)CC2C1C1OC(=O)C(=C)C1C(CC2=C)O	TAS2R44, TAS2R47, TAS2R9
O=C1NS(=O)(=O)c2c1cccc2	TAS2R10, TAS2R5, TAS2R45
OCC1(C)C(O)CCC2(C1CCC(=C)C2C/C=C/1\C(O)COC1=O)C	TAS2R9, TAS2R14
OCC1=CC2OC(=O)C(=C)C2C(CC(=CCC1)C)OC(=O)C(=C)C(CO)O	TAS2R40, TAS2R50, TAS2R14
OOC1CCC2(CC1=CCC1C(C2O)OC(=O)C1=C)C	TAS2R45, TAS2R10, TAS2R43
OCC(=O)C1(O)CCC2C1(C)CC(O)C1C2CCC2=CC(=O)CCC12C	TAS2R42, TAS2R38
N/C(=N\CCCCCC/N=C(/N=C(/Nc1ccc(cc1)Cl)\N)\N)/N=C(/Nc1ccc(cc1)Cl)\N	TAS2R42, TAS2R5, TAS2R44
CCCC(COC(=O)N)(COC(=O)NC(C)C)C	TAS2R3, TAS2R38
COC1=CC(C)C2C(C1=O)(C)C1C(=O)C(=C(C3C1(C(C2)OC(=O)C3)C)C)OC	TAS2R5, TAS2R50
CC1=CCCC2(C)OC2C2C(CC1)C(=C)C(=O)O2	TAS2R7, TAS2R50
NC(=O)c1ccccc1	TAS2R38, TAS2R44
Cn1cnc(c1Sc1ncnc2c1[nH]cn2)[N+](=O)[O-]	TAS2R7, TAS2R38
CCCCCCC/C=C/C(C#CC#CC(C=C)O)O	TAS2R9, TAS2R42
CC(=O)OC(/C=C/C(=O)C(C1C(O)CC2(C1(C)CC(=O)C1(C2CC=C2C1C	TAS2R45, TAS2R43, TAS2R42

C(O)C(=O)C2(C)C)C)C)(O)C)(C)C	
COc1ccc(cc1OC)Cc1nccc2c1cc(OC)c(c2)OC	TAS2R46
COc1ccc2c(c1OC)C(=O)OC2C1N(C)CCc2c1c(OC)c1c(c2)OCO1	TAS2R50, TAS2R42
Oc1ccc2c(c1)oc(=O)c1c2oc2c1ccc(c2)O	TAS2R7, TAS2R40
S=C1NCCN1C	TAS2R5, TAS2R60, TAS2R39
O=C1OCC23C(C1)OC(C2CC(=O)C1(C3CCC2(C31OC3C(=O)OC2c1coc c1)C)C)(C)C	TAS2R1, TAS2R14
CNC(=S)N	TAS2R60, TAS2R44, TAS2R14
Cc1cc(O)nc(n1)S	TAS2R5, TAS2R13
CCc1cnccn1	TAS2R3, TAS2R10
S=CN(C)C	TAS2R40, TAS2R1
S=C1NCCN1	TAS2R48, TAS2R1
CCNC(=S)N	TAS2R16, TAS2R8
CC(=CCC(=O)C1(O)C(=C(C(=O)C1CC=C(C)C)C(=O)C(C)C)O)C	TAS2R16, TAS2R47, TAS2R10
CC(=CCC(=O)C1(O)C(=C(C(=O)C1CC=C(C)C)C(=O)C(C)C)O)C	TAS2R4, TAS2R40
CCC(C(=O)C1=C(O)C(C(=O)C(=C1O)CC=C(C)C)(CC=C(C)C)CC=C(C)C)C	TAS2R16, TAS2R60
NC(=S)Nc1ccccc1	TAS2R16, TAS2R14, TAS2R13
S=C=NCCc1ccccc1	TAS2R13, TAS2R60, TAS2R39
CCC(C(=O)C1=C(O)C(C(C1=O)CC=C(C)C)(O)C(=O)CC=C(C)C)C	TAS2R45, TAS2R44, TAS2R39
CCC(C(=O)C1=C(O)C(C(C1=O)CC=C(C)C)(O)C(=O)CC=C(C)C)C	TAS2R9, TAS2R47
CC(=O)NC(=S)N	TAS2R1, TAS2R50
O=C1CCCCCN1	TAS2R5, TAS2R48, TAS2R60
OCC1OC(OCC2OC(OC(c3ccccc3)C#N)C(C(C2O)O)O)C(C(C1O)O)O	TAS2R40
OCC1OC(Oc2ccc(cc2)O)C(C(C1O)O)O	TAS2R38, TAS2R7
O=C1CC2OCC=C3C4C2C2N1c1ccccc1C12CCN(C1C4)C3	TAS2R9
NC(=Nc1scc(n1)CSCC/C(=N/S(=O)(=O)N)/N)N	TAS2R7, TAS2R16
C=CC/C(=N\OS(=O)(=O)[O-])/SC1OC(CO)C(C(C1O)O)O	TAS2R44, TAS2R3, TAS2R10
C=CCN=C=S	TAS2R48, TAS2R44
OCC1OC(Oc2ccccc2C=O)C(C(C1O)O)O	TAS2R40
OCC1OC(Oc2ccccc2CO)C(C(C1O)O)O	TAS2R38, TAS2R39
C=CC1CN2CCC1CC2C(c1ccnc2c1cc(OC)cc2)O	TAS2R50
CC(=CCC1=C(O)C(C(=O)C(=C1O)C(=O)CC(C)C)(CC=C(C)C)CC=C(C)C)C	TAS2R46
OC(C(=O)c1ccccc1)c1ccccc1	TAS2R48, TAS2R8, TAS2R38
CC(=CCC1=C(O)C(C(=O)C(=C1O)C(=O)CC(C)C)(O)CC=C(C)C)C	TAS2R4, TAS2R48
OCC1OC(Oc2ccccc2)C(C(C1O)O)O	TAS2R50, TAS2R47, TAS2R13
CN(CCC(c1ccccn1)c1ccc(cc1)Cl)C	TAS2R48, TAS2R3, TAS2R49
CN(CCOC(c1ccccc1)c1ccccc1)C	TAS2R1, TAS2R42, TAS2R9
OC(c1ccccc1)(c1ccccc1)CCCN1CCCCC1	TAS2R3
S=C(Nc1ccccc1)Nc1ccccc1	TAS2R44, TAS2R49, TAS2R45
C=CS(=O)C=C	TAS2R42
OC(=O)c1ccccc1Nc1cccc(c1)C(F)(F)F	TAS2R42, TAS2R7, TAS2R39
Fc1ccc(cc1)C(=O)CCCN1CCC(CC1)(O)c1ccc(cc1)Cl	TAS2R60, TAS2R39

[O-]C(=O)c1ccccc1	TAS2R43, TAS2R8, TAS2R42
OCCc1sc[n+](c1C)Cc1cnc(nc1N)C	TAS2R8, TAS2R49
OCC(C(c1ccc(cc1)[N+](=O)[O-])O)NC(=O)C(Cl)Cl	TAS2R40, TAS2R14, TAS2R5
O=C(c1cc(O)c(c(c1)O)O)O[C@@H]1[C@@H](OC(=O)c2cc(O)c(c(c2)O)O)[C@@H](OC([C@@H]1OC(=O)c1cc(O)c(c(c1)O)O)OC(=O)c1cc(O)c(c(c1)O)O)OC(=O)c1cc(O)c(c(c1)O)O	TAS2R4, TAS2R49, TAS2R46
CCC1CN2CCc3c(C2CC1CC1NCCc2c1cc(OC)c(c2)OC)cc(c(c3)OC)OC	TAS2R13
OCC1OC(Oc2ccccc2CO)C(C(C1O)O)O	TAS2R45, TAS2R7, TAS2R47
CC1CC(C)C(=O)C(C1)C(CC1CC(=O)NC(=O)C1)O	TAS2R9, TAS2R43, TAS2R46
COc1cc2c(cc1OC)N1C3C42CCN2C4CC4C3C(CC1=O)OCC=C4C2	TAS2R43, TAS2R8, TAS2R47
O=C1CC2(C(C1C)C2)C(C)C	TAS2R4, TAS2R48, TAS2R38
Nc1ccc(cc1)S(=O)(=O)c1ccc(cc1)N	TAS2R46, TAS2R47, TAS2R14
O=C1OC2C(C1C)(O)C13C4(C2)C(OC3=O)CC(C24C(O1)OC(=O)C2O)C(C)(C)C	TAS2R8, TAS2R16, TAS2R9
c1ccc2c(n1)c1ncccc1cc2	TAS2R14, TAS2R38, TAS2R45
CC[N+](Cc1ccccc1)(CC(=O)Nc1c(C)cccc1C)CC	TAS2R49, TAS2R48, TAS2R1
CC(=O)OC(/C=C/C(=O)C(C1C(O)CC2(C1(C)CC(=O)C1(C2CC=C2C1C=C(O)C(=O)C2(C)C)C)C)(O)C)(C)C	TAS2R50, TAS2R45, TAS2R46
OC(COc1cccc2c1c(=O)cc(o2)C(=O)O)COc1ccc2c(c1)c(=O)cc(o2)C(=O)O	TAS2R3, TAS2R40
CC(=CCc1c(O)cc(c2c1OC(CC2=O)c1ccc(cc1)O)O)C	TAS2R43, TAS2R10
CC(=C)C1C2OC(=O)C1C1(C3(C2OC(=O)C23C(C1)O2)C)O	TAS2R13, TAS2R49, TAS2R48
Oc1cc(O)c2c(c1)O[C@@H]([C@H]([C@@H]2c1c(O)cc(c2c1O[C@H](c1ccc(c(c1)O)O)[C@H]([C@@H]2c1c(O)cc(c2c1O[C@@H]([C@H](C2)O)c1ccc(c(c1)O)O)O)O)O)O)c1ccc(c(c1)O)O	TAS2R45, TAS2R14
C[C@@H]1C(=O)O[C@H]2[C@H]1CC[C@@]1(C2=C(C)C(=O)C=C1)C	TAS2R16, TAS2R5, TAS2R47
C/C/1=C\[C@H]2OC(=O)C(=C)[C@@H]2CC/C(=C/CC1)/C	TAS2R60, TAS2R8, TAS2R1
Oc1cc2O[C@H](c3ccc(c(c3)O)O)[C@H](Cc2c(c1)O)O	TAS2R39
OC[C@H]1O[C@@H](Oc2cc3c(O)cc(cc3[o+]c2c2cc(OC)c(c(c2)OC)O)O)[C@@H]([C@H]([C@@H]1O)O)O	TAS2R13, TAS2R4, TAS2R49
NCCS(=O)(=O)O	TAS2R43, TAS2R4, TAS2R39
O=CC1(CC(c2cocc2)O)C(C)C(OC(=O)C)CC2(C1CCC(C2(C)O)O)C	TAS2R50, TAS2R4
O=c1ccc2c(o1)cccc2	TAS2R38, TAS2R39
Cn1cnc2c1c(=O)n(C)c(=O)n2C	TAS2R3, TAS2R13, TAS2R44
[O-]S(=O)(=O)NC1CCCCC1	TAS2R45, TAS2R14, TAS2R7
COc1cccc2c1cc([N+](=O)[O-])c1c2c2OCOc2cc1C(=O)O	TAS2R49, TAS2R13
CC1=CC2OC(=O)C(=C)C2CCC(=C)C(CC1)O	TAS2R40, TAS2R45, TAS2R44
CC1=CCC23C1C1OC(=O)C(C1CCC3(O2)C)C	TAS2R50, TAS2R8
CC1=CCC23C1C1OC(=O)C(=C)C1CCC3(O2)C	TAS2R48, TAS2R3, TAS2R8
CC1C(=O)OC2C1CCC(C1C2=C(C)C2C1C1C3C2(C(=C1)C)C1OC(=O)C(C1CCC3(C)O)C)(C)O	TAS2R5, TAS2R1, TAS2R39
COc1ccc2c(c1)C13CCCCC3C(C2)N(CC1)C	TAS2R14
COc1cc(O)c(c(c1C(=O)/C=C/c1ccc(cc1)O)O)CC=C(C)C	TAS2R9, TAS2R49, TAS2R42

COc1cc(O)c(c2c1C(=O)CC(O2)c1ccc(cc1)O)CC=C(C)C	TAS2R3
CC(=CCC1=C(O)C(C(=O)C(=C1O)C(=O)C(C)C)(CC=C(C)C)CC=C(C)C)C	TAS2R5
CC(=CCC1=C(O)C(C(=O)C(=C1O)C(=O)C(C)C)(O)CC=C(C)C)C	TAS2R38
CCC(C(=O)C1=C(O)C(C(=O)C(C1=O)CC=C(C)C)(O)CC=C(C)C)C	TAS2R13, TAS2R10
OCC1OC(OC2OC=C3C(C2C=C)CCOC3=O)C(C(C1O)O)OC(=O)c1c(O)cc(cc1c1cccc(c1)O)O	TAS2R16, TAS2R10
COC(=O)C1C(O)CCC2C1CC1N(C2)CCc2c1[nH]c1c2cccc1	TAS2R3
CC(CC(=O)C1=C(O)C(C(C1=O)CC=C(C)C)(O)C(=O)CC=C(C)C)C	TAS2R4, TAS2R7
CC(=O)c1cnccn1	TAS2R47
O=C1OC(=O)C2(C(C1CC2)(C)C)C	TAS2R46
Oc1ccc(cc1)[C@H]1COc2c(C1)ccc(c2)O	TAS2R7, TAS2R40, TAS2R44
Oc1ccc(cc1)c1coc2c(c1=O)c(O)cc(c2)O	TAS2R60, TAS2R4, TAS2R49

Table S6. The in-house library composed of 220 chemical probes in the SMILES format.

Num.	Fragments
1	ON1C(C)(C)CC(CC1(C)C)N=C=S
2	FC1(F)C(F)(F)OS1(=O)=O
3	c1(noc(c1)c1ccc(cc1)Br)C(=O)O
4	N#CCc1c(C)c(C)c(c(c1C)C)C
5	O/N=C(\Cc1ccccc1)/C
6	C[Si]1(C)N[Si](C)(C)N[Si](N1)(C)C
7	C[Si](P([Si](C)(C)C)[Si](C)(C)C)(C)C
8	N#CCCSc1sc(=O)sc1SCCC#N
9	c1(csc(n1)C)C(=O)OCC
10	FS(=O)(=O)C(F)(F)F
11	Sc1nc(=O)n2c(n1)ccc(c2)Cl
12	C12(CC(=O)N(C(=O)C1)CCC)CCCC2
13	N#CC1=CC[C@@]23[C@@]1(CCC2)C(=CC3)C#N
14	FCCN1CCN(CC1)C(=O)Cl
15	O[C@H]1C[C@@H](C)[C@H]2[C@H](C(=C1)C)CC(=C(C)C)C2
16	Nc1nc(N)nc(n1)N
17	CN([Si](N(C)C)(N(C)C)C)C
18	[C@]12([C@@H](O1)C[C@@H](CC2)C(=C)C)C
19	N(CCCCCC)(CCCCCC)N=O
20	C(=O)(N1CCN(CC1)CC=C)CC(=O)NN
21	Fc1ccc2c3c1ccc1c3c(cc2)ccc1
22	CCCN1CCC(=O)CC1
23	c12c([nH]nc1O)cc(cc2Cl)I
24	FC(C1=[NH]S(=O)(=O)NC(=C1)C)(F)F
25	Fc1ccc(c2c1cn[nH]2)I
26	c1nc(=O)c(c[nH]1)Br

27	COc1ccc(cc1)c1ncc[nH]1
28	C[SiH](C(C)(C)C)C(C)(C)C
29	COCCN1CCN(CC1)CC[N]#C
30	c1(c(c(cc(c1)/C=C\C(=O)O)OC)OC)S(=O)(=O)Nc1ccc(cc1)C(=O)O
31	c12c([nH]c(c1)C(=O)OC)cccc2
32	c1(cc(cc(c1)C(C)C)C(C)C)C(C)C
33	O[C@H]1CCC(=C1)I
34	C1(=O)[C@H]2[C@@H](C1)C=CC2
35	CCN1CCOCC1
36	CCOP(OP(OCC)OCC)OCC
37	c1(n2cccc2)ccc(cn1)I
38	FC(=C(C(F)(F)F)Br)F
39	CC([NH2]NC(C)(C)C)(C)C
40	COP(=O)(SCc1nc(N)nc(n1)N)OC
41	COCCOCOCCOC
42	C12([C@@H](O1)C(=O)OC)CCCCC2
43	CN(/C=N/c1cc[nH]c(=O)n1)C
44	COC1CSC1
45	Cl[C@H]1CC[C@@H]2[C@@H](C1)NON2O
46	Fc1ccc(c2c1cn[nH]2)Br
47	BrC[C@@H]1CC[C@H](O1)c1ccncc1
48	O=CCC(C(C(F)(F)F)(F)F)(F)F
49	n12c(=NCC1)ssc2=S
50	Brc1n[nH]cn1
51	CC(SSC(C)(C)C)(C)C
52	CC1CCN(CC1)CCCS(=O)(=O)O
53	o1nc2c(n1)nc1c(n2)[nH]c2c([nH]1)c1nsnc1cc2
54	C12=CNCCC[C@@H]1C=C(C=C2)OC
55	C\1=C/C=N\[C@H]2C[C@@H]2N/C=C/C=N\[C@@H]2[C@@H](N1)C2
56	COP(=O)OC
57	N#Cc1ncn2c1nnn(c2=O)C
58	O=S1(=O)CC=CC1
59	c1(/C=C\2/C(=C(C=N2)C)C)c(c(c[nH]1)C)C
60	CSc1scc(c1)/C=N/NC(=O)N
61	O=CN1CCN(CC1)C=O
62	Cc1cc(C)nc(n1)Nc1ccc(cc1)OC(F)(F)F
63	ICC(C(F)F)(F)F
64	C[C@H]1CCCC[C@H]1N[C@H]1CCS(=O)(=O)C1
65	C1CCCC(CC1)NC1CCOCC1
66	c1(nnco1)SCC(=O)N
67	OCCCCCCCCCCCCO
68	C1=CC=CC=c2c(=C1)nc1ccccc1n2
69	NCCOCCN
70	c1(c(ccn(c1=O)C)OC)C#N

71	S(Cc1nnn[nH]1)Cc1nnn[nH]1
72	CN1CCN(CC1)C(=O)NC(C)(C)C
73	CN1CCN(CC1)C(=O)N
74	FC(C(Cl)(Cl)Cl)(C(Cl)(F)F)F
75	OC(=O)Cn1nc2c(n1)cccc2
76	ClC(=O)CCC[Si](Cl)(Cl)C
77	c1(c2ccc(cc2)OC)ccc(cc1)OC
78	Brc1ccc(cc1)c1cccc(c1)Cl
79	CO[Si](OC)(OC)OC
80	BrCc1onc(c1)Br
81	c1(c2cnccn2)ccno1
82	NN1C(=S)S[C@H]2[C@@H]1CS(=O)(=O)C2
83	Nc1ccc2=c3c(=c2c1)cccc3
84	N#Cc1c(N)onc1c1ccc(cc1)Br
85	O=Nc1c(N)nc(nc1N)N
86	Cn1c(COc2ccccc2)nnc1S
87	N#CCCSSCCC#N
88	CCCCCCCC[C@H]1CO1
89	c1(c2ccccc2)nc2c(s1)cccc2
90	CCSc1nccc(c1C#N)c1ccc(cc1)Cl
91	C=CS(=O)(=O)O
92	O=C1CNc2c(N1)cnc(n2)N
93	NC[C@@H]1N[C@H](CCl)CN([C@H]1N)O
94	N[C@]12C[C@H]3C[C@@H](C2)C[C@@H](C1)C3
95	c1(C(=O)NC2CC2)cn(nc1)CCC(=O)O
96	OC(C#CC(C)(C)C)C#CC(C)(C)C
97	C(C(=C/C=N/C(C)(C)C)C[Si](C)(C)C)[Si](C)(C)C
98	Fc1ccc2c(c1)c(=O)n1c(n2)CCCCC1
99	C1CC(C1)CNC[C@H]1CCCO1
100	ClCCNC(=O)N(CCCl)CCCl
101	O=Cc1ccccc1OC
102	Clc1ncc(nc1)OC
103	n1(c2ccc(cc2)C)c(ccc1)C=O
104	ClC[C@@H]1CN(O)[C@H]([C@@H](N1)C#N)N
105	CCCn1ccc(n1)C=O
106	c12c3c([nH]c1C(=O)CCC2)cccc3
107	Nc1ccc(c(c1)Cl)Oc1cc(C)cc(c1)C
108	c1(c(n[nH]c(=O)n1)C)N/N=C/c1ccccc1
109	c12n(c(nc1cccc2)CC)CC(=O)O
110	NCCc1c(C)nn(c1N1CCOCC1)C
111	COCCN(CCCl)CCCl
112	COS(=O)(=O)OC
113	n1ccc2[nH]cc(c2c1)Br
114	BrN1C(=O)CCC1=O

115	c1(/C(=C/C=O)/Cl)ccc(cc1)F
116	OCc1[nH]c2c(n1)cc1c(c2)[nH]cn1
117	N[C@H]1CCN(CC1)C(=O)c1ccccc1
118	C[Si]1(C)CCC1
119	c1(n(nnn1)CCCC)NCc1ccccc1
120	O=C=NC[C@@H]1CCC[C@H](C1)CN=C=O
121	c1(C(=O)CSc2[nH]c(nn2)N)ccccc1
122	c1ccc(cc1)c1nc2c([nH]1)cccc2
123	NN1C(=O)CCC1=O
124	C(#CC=O)[Si](C)(C)C
125	c1ccc2c(c1)c1ccccc1c1c2cccc1
126	N#CC1=CC[C@]2([C@]1(CC)CC=C2C#N)C
127	CC(=O)Nc1nc2c(s1)ccc(c2)Br
128	N#CC(=C(C#N)C#N)C#N
129	Sc1[nH]c(=O)c2c(n1)n(nc2)c1ccccc1
130	C1CCCCC(CC1)NC1CCOCC1
131	c1(c2ccc(cc2)OC)nc(ncc1)N
132	Brc1ccc(s1)CCc1c[nH]cn1
133	CCSc1nc(N)cc(=O)[nH]1
134	n1(c(cc(cc1=O)C)C)N
135	N[C@@H]1CONC1=O
136	c12c([nH]c(=S)nc1)CC[C@H](C2)N
137	N/N=c/1\sc2c([nH]1)c(Cl)cc(c2)Br
138	c12c(nc([nH]1)CCN)cccc2
139	OS(=O)(=O)c1ccccn1
140	[Si](CC)(F)(F)F
141	Sc1nnc(n1N)S
142	c1([C@@H](CN2CCN(CC2)C)N)ccccc1
143	CCCCn1ccnc1CNCCOC
144	[C@@]12(c3c([C@H](C1(C)C)CC2)nnc(n3)S)C
145	Cc1cc(Nc2ccccc2)c2c(n1)c1ccccc1cc2
146	OS(=O)(=O)CCS(=O)(=O)O
147	NC(=N)N/N=C/1\C=CC(=O)C=C1
148	C1(=C(N[C@H](N1)C=C)C)C(=O)C
149	BrC1=CCCS1(=O)=O
150	C[C@]1(CSC(=O)S1)/C=N/O
151	FC(c1cc(Cl)c(cc1Cl)C(F)(F)F)(F)F
152	ClC(=O)CCc1c(F)c(F)c(c(c1F)F)F
153	ClC(=O)N1CCCCC1
154	CCCCOP(=O)(OCCCC)OCCCC
155	Fc1ccc(c(c1)F)Oc1ccc(c(c1)C(F)(F)F)N
156	Brc1csc(c1)Cl
157	CC(C#C[Si](C)(C)C)(C)C
158	N1[C@@H](O)CCCC1

159 Clc1cc(nc(n1)C(F)(F)F)N1CCOCC1
160 C1(C([C@@H]2[C@@H]3[C@H]1[C@@H]1[C@H]2[C@@H]1C3)(C#N)C#N)(C#N)C#N
161 c1(ccc(cc1)C(=O)O)c1nc(no1)C
162 c12c(ccc(c1)C(F)(F)F)[nH]c(n2)C
163 ClCc1ccon1
164 ON1C[C@@H](C)[C@H]([C@@H](C1)C)[N](=O)O
165 N=C(Nc1ccccc1)NC(=N)N
166 C1CN=C(N1)CC2=CC=CC=C2
167 C=C/C=C/C(C(F)(F)F)(C(F)(F)F)C(F)(F)F
168 ClCCn1nnc2n(c1=O)cnc2C(=O)N
169 OC[C@H]1CN1[C@@H](c1ccccc1)C
170 O=Cc1c2=Nc3c(c2cc2=c4c(=Nc12)cccc4)cccc3
171 NCCCCCC(=O)O
172 CCCCCC/C=C/CCCCCCCC(=O)Cl
173 c1ccc(cc1)[C@@H]1NN=C(C1)c1ccccc1
174 CN(S(=O)(=O)N(C)C)C
175 COc1nc(nc(n1)N)NC(C)(C)C
176 C12(C(=O)OC[C@H](N1)CC)CCCC2
177 CSC[C@@H]1CO1
178 Cc1cc(Br)c(=O)[nH]n1
179 P(=O)(OCCCC)(OCCCC)CCCC
180 NCc1nnc(o1)C1CCCC1
181 CN(S(=O)(=O)N1CCNCC1)C
182 [C@@H]1([C@H]([C@H](C=C[C@@H]1O)O)O)O
183 c12c3c(ccc1oc(n2)C)nsn3
184 CC(CC([NH3])(C)C)([NH3])C
185 c12c(sc(c1C)C=O)scc2C
186 OC(=O)Cn1nnc(n1)N1CCOCC1
187 O=CN(CCC#N)CCC#N
188 CO[Si](Cl)(C)C
189 CCOc1ccc(cc1Cl)F
190 c12c3c([nH]c1cnc(c2)C(=O)OC)cccc3
191 c1(c(cc(cc1)Br)Br)OCC#N
192 c1(c(n[nH]c1N)C)Cl
193 FC(C(C(C([C@]1(F)OC1(F)F)(F)F)(F)F)(F)F)F
194 n1(c2nc(cc(n2)C)C)nc(cc1C)C
195 C=CCSc1nc2cc(C)ccn2c(=O)n1
196 OC(=O)c1c(C)cc(cc1C)C(C)(C)C
197 c12c(C(=O)c3c(C1=O)cccc3)ccc(c2)C
198 CN(C(=O)N(C)C)C
199 Sc1nn2c(s1)nnc(c2=O)C(C)(C)C
200 c1(c2cc(c(cc2)Cl)Cl)cc(c(cc1)Cl)Cl
201 [C@H]12[C@H](C(=O)OC1=O)CC=CC2

202	ClC(P(Cl)(Cl)(Cl)Cl)(Cl)Cl
203	c1ccc2c(c1)c1c3c2cccc3cc2c1cccc2
204	c1csc(c1)c1cccs1
205	O=C(CN1CCNCC1)NC1CC1
206	c1(c2n(c(=O)cc1O)cccc2)C(=O)OC
207	Clc1ncc(nc1)Cl
208	CCC(NC(CC)(C)C)(C)C
209	CCCN1CCC(CC1)NC[C@H]1CCCO1
210	S(=O)(=O)(c1ccc(N2CCNCC2)cc1)N
211	CCCCCCCCCCCCCCP(=O)(O)O
212	S=C=Nc1ccc(cc1C)C(C)(C)C
213	ClP1OCCO1
214	CSCC[C@H]1NCCNC1=O
215	n1(c(=S)[nH]nc1)N1CCCCC1
216	Sc1nn2c(s1)nnc(c2=O)C
217	C1CCc2c(C1)c1CCCc3c1c1c2CCCc1cc3
218	C1=CC=C(C(=C1)C(=O)N)O
219	N(=C=NC(C)(C)C)C(C)(C)C
220	Cc1ccc(cc1)OCc1nnc(s1)N

2 FIGURES

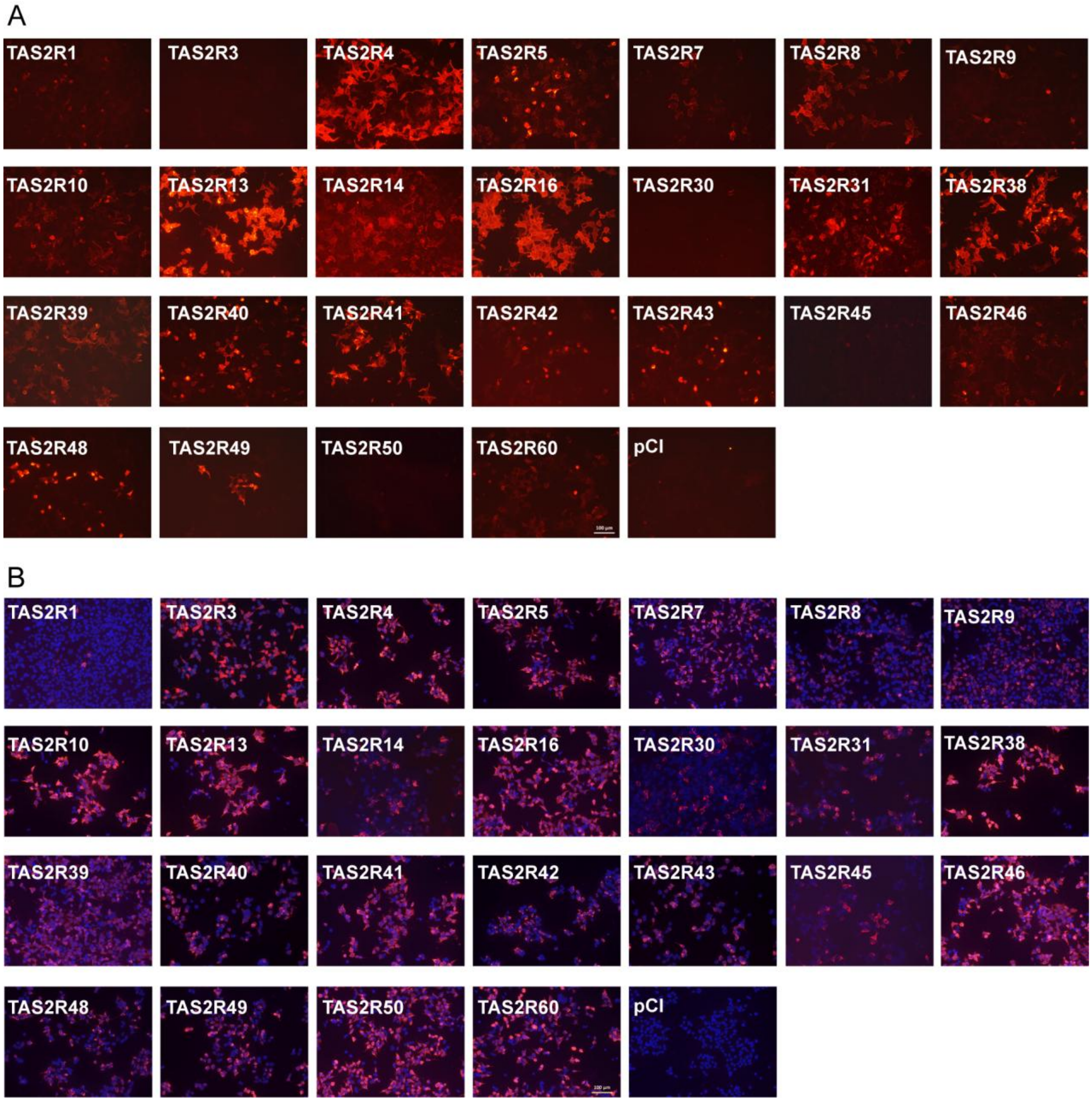

Figure S1. The (A) cell-surface expression and (B) total expression of all TAS2R constructs when transfected into HEK293T cells. For optimal expression, RTP3 were co-transfected with TAS2R7, TAS2R8, TAS2R9, TAS2R40, TAS2R43, and TAS2R49, and RTP4 were co-transfected with TAS2R3, TAS2R42, TAS2R48, and TAS2R60. (Scale bar = 100 µm).

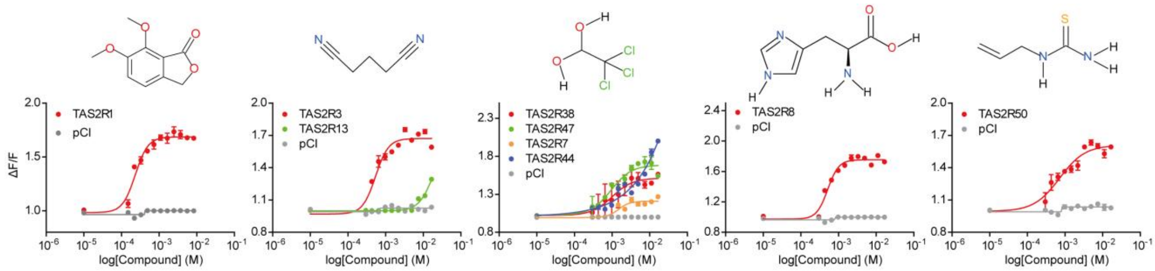

Figure S2. The positive-control dose-response curves of 9 TAS2Rs that tested negative in our system (Ji et al. 2015). *y*-axis represents ΔF/F ± SEM (*N* = 2). “pCI” denotes the response of mock-transfected cells to each compound. The structures of the compounds tested are shown above each graph.
